# Conspiracy theories and misinformation about COVID-19 in Nigeria: Implications for vaccine demand generation communications

**DOI:** 10.1016/j.vaccine.2022.02.005

**Published:** 2022-03-18

**Authors:** Chizoba Wonodi, Chisom Obi-Jeff, Funmilayo Adewumi, Somto Chloe Keluo-Udeke, Rachel Gur-Arie, Carleigh Krubiner, Elana Felice Jaffe, Tobi Bamiduro, Ruth Karron, Ruth Faden

**Affiliations:** a Johns Hopkins University Bloomberg School of Public Health, Baltimore, MD, United States; b Direct Consulting and Logistics Limited Abuja, Federal Capital Territory, Nigeria; c Women Advocates for Vaccine Access Abuja, Federal Capital Territory, Nigeria; d Berman Institute of Bioethics, Johns Hopkins University, Baltimore, MD, United States; e University of North Carolina, School of Medicine, Chapel Hill, NC, United States

**Keywords:** Conspiracy theory, Misinformation, COVID-19 vaccines, Risk communication, Demand generation communication, Nigeria, FCT, Federal Capital Territory, FGD, Focus Group Discussion, HCWs, Healthcare Workers, KII, Key Informant Interviews, LGA, Local Government Area, LMIC, Low- and Middle-Income Countries, NCDC, Nigeria Centre for Disease Control, NPHCDA, National Primary Health Care Development Agency, PHC, Primary Health Care, RI, Routine Immunization, WHO, World Health Organization

## Abstract

**Introduction:**

COVID-19 vaccine hesitancy is a worldwide phenomenon and a serious threat to pandemic control efforts. Until recently, COVID-19 vaccine hesitancy was not the cause of low vaccine coverage in Nigeria; vaccine scarcity was the problem. As the global supply of COVID-19 vaccines improves in the second half of 2021 and more doses are deployed in Nigeria, the supply/demand dynamic will switch. Vaccine acceptance will become a key driver of coverage; thus, amplifying the impact of vaccine hesitancy. Conspiracy theories and misinformation about COVID-19 are rampant and have been shown to drive vaccine hesitancy and refusal. This study systematically elicits the misinformation and conspiracy theories circulating about COVID-19 among the Nigerian public to understand relevant themes and potential message framing for communication efforts to improve vaccine uptake.

**Methods:**

From February 1 to 8, 2021, we conducted 22 focus group discussions and 24 key informant interviews with 178 participants from six states representing the six geopolitical zones. Participants were purposively selected and included sub-national program managers, healthcare workers, and community members. All interviews were iteratively analyzed using a framework analysis approach.

**Results:**

We elicited a total of 33 different conspiracy theories or misinformation that participants had heard about the COVID-19 virus, pandemic response, or vaccine. All participants had heard some misinformation. The leading claim was that COVID-19 was not real, and politicians took advantage of the situation and misused funds. People believed certain claims based on distrust of government, their understanding of Christian scripture, or their lack of personal experience with COVID-19.

**Conclusions:**

Our study is the first to report a thematic analysis of the range of circulating misinformation about COVID-19 in Nigeria. Our findings provide new insights into why people believe these theories, which could help the immunization program improve demand generation communication for COVID-19 vaccines by targeting unsubstantiated claims.

## Highlights

• First thematic analysis of circulating misinformation about COVID-19 in Nigeria.
• Found universal exposure to misinformation on COVID-19 and the vaccine.
• Top false claim was that COVID-19 is fake and used by politicians to misuse funds.
• Tracking public opinion on COVID-19 will help efforts to counter vaccine hesitancy.
• Adaptive strategies and messages will counter COVID-19 vaccine misinformation.

## 1 Introduction

Vaccination is a centerpiece of the global response to the severe acute respiratory syndrome coronavirus-2 (SARS-CoV-2), a virus that causes coronavirus disease 19 (COVID-19). The SARS-CoV-2 pandemic has resulted in more than 275 million cumulative cases and 5.3 million deaths as of December 22, 2021 [1]. COVID-19 vaccine hesitancy is a worldwide problem worsened by misinformation and conspiracy theories about the disease and the vaccines [2], [3], [4]. These false narratives have spread rapidly across the globe through ubiquitous internet and social media platforms. The World Health Organization (WHO) describes the overwhelming volume and circulation of false information about the SARS-CoV-2 pandemic as an “infodemic” that seriously threatens global and local efforts to end the pandemic.

Nigeria was among the first countries in sub-Saharan Africa to identify COVID-19 cases [5]. Since the Federal Ministry of Health reported its first index case on February 27, 2020 [6], Nigeria has deployed public health and social measures such as banning public gatherings and restricting movement and businesses to curb the virus transmission. As of December 23, 2021, Nigeria has 231,413 cases across all states, of which 211,853 were discharged and 2991 confirmed dead [7]. Since the pandemic, males are mostly affected than females, with a 60: 40 case ratio and 74:26 death ratio [7]. Individuals 50 years and above account for 70% of the mortality burden [7]. All states in the country are affected, but most cases are reported in Lagos and the Federal Capital Territory (FCT) [7]. While recent data trends have suggested a continuous decrease in the number of cases, the spread of the pandemic, the second wave in December 2020 [8], and non-adherence to non-pharmaceutical preventive measures underscored the need for an effective vaccine. Vaccination with safe and effective COVID-19 vaccines is critical for Nigeria’s strategy to interrupt the COVID-19 pandemic and curb transmission.

Historically, Nigeria has grappled with highly disruptive vaccine hesitancy and refusal resulting from negative rumors and loss of public confidence. For example, in 2003, a rumor that the oral polio vaccine contained porcine material and sterilizing agents led to a boycott of polio vaccination in Northern Nigeria. Following this vaccine boycott, an upsurge in polio cases resulted in wild-polio re-infection in 20 countries across Africa and Asia [9], [10], [11] and retrogression of polio eradication efforts in Nigeria and the African region. In 2017, another rumor broke out in the country’s Southeastern part, purporting that the Nigerian military was “vaccinating” school children with the monkeypox virus [12]. This false story led panicked parents to withdraw their children from school and, uncharacteristically, refuse to take them for vaccination. Soon after that, vaccination rates dropped in that part of the country – a region that previously had some of the highest vaccine uptake rates in the country.

Early vaccine procurement, domestic production of vaccines, the severity of the pandemic, a country’s health infrastructure, and vaccine acceptance are significant determinants of COVID-19 vaccine coverage [13]. However, evidence has shown that widespread vaccine acceptance is crucial for achieving sufficient immunization coverage [14]. Until recently, hesitancy surrounding COVID-19 vaccines was not the top programmatic bottleneck to widespread use of COVID-19 vaccines in Nigeria and other low- and middle-income countries (LMICs) because the vaccines were in short supply globally. Thus, like other LMICs, Nigeria introduced COVID-19 vaccines with barely enough doses to cover 1% of the population, with vaccine demand far outstripping vaccine supply. As the global supply of COVID-19 vaccines improves in the second half of 2021 and more doses become available and deployed in LMICs, demand and acceptance, along with absorption capacity, will become the top coverage drivers. Therefore, the need to address vaccine hesitancy will become even more critical and urgent. For example, Nigeria received 40 million COVID-19 vaccine doses in August 2021 and commenced the second vaccination phase [15]. With the planned deployment of millions of vaccine doses, demand generation activities must take center stage, and part of this effort must include practical strategies to address and debunk misinformation and conspiracy theories about COVID-19 and COVID-19 vaccines. We use the term misinformation to denote false or inaccurate information or communication [16]. We consider conspiracy theories as a particular kind of false information in which the creators of conspiracy theories attribute the root causes of events or trends to shadowy networks or cabals carrying out secret plots to control, undermine or exploit the public, governments, or institutions [17]. Conspiracy theories reject the standard explanations for significant phenomena and instead credit covert actors and manipulators.

For a vaccination program to succeed, implementers must first understand and contextualize the range, breadth, and depth of circulating misinformation and conspiracy theories. It is also important to understand the patterns and drivers of belief in these conspiracy theories and how these beliefs affect vaccination intentions in the communities and among different demographic groups. This study was designed to systematically elicit the circulating COVID-19 misinformation and conspiracy theories that the Nigerian public had heard or read. We also sought to describe the themes and patterns of beliefs associated with these narratives to produce evidence to guide communication efforts towards reducing vaccine hesitancy and improving uptake. At the time of data collection, COVID-19 vaccines had not yet been deployed in the country, although the plans for introduction were advanced.

## 2 Methods

### 2.1 Study setting

We conducted the study in six of the 36 states in Nigeria: Cross Rivers, Ebonyi, Gombe, Kano, and Lagos states, including the FCT. These states were selected to represent each of the six geopolitical zones in the country and reflect states with a low, medium, and high burden of COVID cases relative to the national picture. See Supplementary Materials 1 and 2 for details. Following this, we selected two local government areas (LGAs) /sub-districts in each state/district to ensure a mix of settings that represent demographic, logistical, and epidemiologic diversity, as follows: 1) rural, urban, and semi-urban, 2) proximal and distal to the state capital, 3) SARS-CoV-2 transmission hot and cold spots, and 4) presence of a research partner organization to support data collection. A total of 12 LGAs were selected and visited.

### 2.2 Study design

This paper only presents the qualitative arm of a mixed-methods study that used qualitative and quantitative approaches to examine the phenomenon of misinformation and conspiracy theories about COVID-19 and COVID-19 vaccines in Nigeria. We conducted focus group discussions (FGDs) and key informant interviews (KIIs) to examine the misinformation and conspiracy theories surrounding the SARS-CoV-2 pandemic.

### 2.3 Study participants and sampling

We purposively selected participants for both the FGDs and KIIs from routine immunization (RI) program personnel and community members. The RI program personnel were program managers at the state and LGA levels and healthcare workers (HCWs) at primary health care (PHC), secondary, or tertiary care levels. Program managers and HCWs were aged 18 years and above and occupied their current roles for at least a year. Community members included pregnant women, parents of children under five years of age, and Christian and Muslim religious leaders. We also selected male and female youths (18 – 24 years), adults (25 – 49 years), and older adults (>=50 years). To be eligible for the FGDs, community participants had to be 18 years or older and residents in the community for at least one year to ensure familiarity with community norms. For the FGDs and KIIs, we excluded individuals screened and assessed to have COVID-19 symptoms. Community leaders identified FGD participants with the help of local HCWs or immunization program managers in the state. The State Primary Health Care Board and the State Emergency Routine Immunization Coordinating Centre identified the KII participants, most of whom were immunization program managers. All the persons nominated for the interviews agreed to participate in the study.

In total, we conducted 22 FGDs and 24 KIIs among 178 people from February 1 to 8, 2021. All 24 KIIs were conducted with state and LGA program managers and HCWs. Sixteen FGDs were conducted with community members, three with religious leaders, and three with HCWs. At least three KIIs and three FGDs were conducted in each of the six states. Supplementary Materials 3 provides a complete list of the participants, interviews conducted, and the number of participants interviewed.

### 2.4 Data collection

Our trained data collectors conducted the FGDs and KIIs using pre-tested semi-structured interviews and discussion guides and the tracking sheet to collect participants’ socio-demographic information. After obtaining verbal consent, all interviews (FGDs and KIIs) lasted between 45 and 60 min. While the KIIs were conducted at the participant’s home or a convenient location, the FGDs were conducted outdoors. Interview guides were in English. However, data collectors conducted interviews in local languages to ease communication and understanding. Interviews conducted in local languages were transcribed in the language by external vendors and back-translated to English for validation. We provided FGD participants at the community level one thousand naira only ($2.6) for logistics during the study data collection period.

Data collection teams followed all COVID-19 preventive guidelines such as masking, hand washing with soap and water, and maintaining 2 m distance between each other before and during the interviews. The researchers provided the teams’ supervisors with portable infrared thermometers for daily temperature screening of data collectors during the fieldwork. At the start of each day’s activities, the team supervisor verbally screened data collection teams for COVID-19 symptoms. Likewise, data collectors conducted verbal symptom screening and temperature checks on participants before interviews to rule out suspected COVID-19 cases. There were no suspected COVID-19 cases amongst the field staff and the participants during the data collection period.

### 2.5 Data analysis

We analyzed the qualitative interviews iteratively using a Framework Analysis approach, adapted from the WHO behavioral and psychosocial determinants of vaccine acceptance framework [18] and Gale et al. framework methodology [19]. First, we audio-recorded all interviews and transcribed them verbatim. Three study team members read the transcripts for content comprehension and familiarization. Then, ten transcripts were randomly selected and coded independently by three team members. The team members then compared the codes across the three coders to generate the first unified set of codes. Third, the study team used these unified codes to create the initial analytical framework. Upon reading subsequent transcripts, the initial analytical framework was revised iteratively until we generated no new codes. Recurrent codes, similar or related codes, were grouped into defined categories, culminating into our final framework, consisting of 68 unique codes clustered into 31 categories. These codes and categories were used to abstract data from each transcript.

The study team generated themes by reviewing and collating categories and their associated patterns. We further reviewed each theme to ensure an accurate representation of the data and included a brief explanatory description of its meaning. Subsequently, connections across participants, categories, and themes were drawn by reviewing the summary spreadsheet of abstracted data. We also analyzed the themes and categories for geographic patterns. We anonymized the quotes for confidentiality. It is worth noting that this process was influenced both by the research objectives and by new concepts generated inductively from the data. Supplementary Material 4 presents the analytical framework used in this study. The completed interview tracking sheets were entered into Microsoft Excel and analyzed descriptively for frequencies and percentages.

### 2.6 Ethics approval

We obtained ethics approval for the study from the National Health Research Ethics Committee of Nigeria (NHREC/01/01/2007-05/11/2020) and the Johns Hopkins University Institutional Review Board (IRB00014705). We also obtained additional state-level ethics approval from the Health Research Ethics Committees in Cross River, Ebonyi, Gombe states, and the FCT as was required. These research ethics committees reviewed and approved the oral consent script used to solicit consent from all individuals interviewed.

## 3 Results

### 3.1 Socio-demographic characteristics of study participants

We conducted 46 individual interviews and group discussions with 178 participants, the majority of whom were community members 134 (75%), females 97 (54%), urban residents 122 (69%), and individuals with formal education 146 (82%). Participants’ ages ranged from 20 to 80 years, with a mean age of 40 ± 12.8 years (Table 1).

**Table 1:** Socio-demographic characteristics of participants.

**Characteristics**	**Number** **(n = 178)**	**Percentage (%)**
***Participant type***		
Community members	134	75%
Program managers	32	18%
Health workers	12	7%
***Sex***		
Male	81	46%
Female	97	54%
***Residence***		
Urban	122	69%
Rural	56	31%
***Attended formal education***		
Yes	146	82%
No	32	18%
***Highest level of formal education completed***		
None	32	18%
Primary School	15	8%
Secondary School	55	31%
Graduate	62	35%
Post-Graduate	14	8%

### 3.2 Prevalent conspiracy theories and misinformation about COVID-19 and the vaccines

**Fig. 1:**
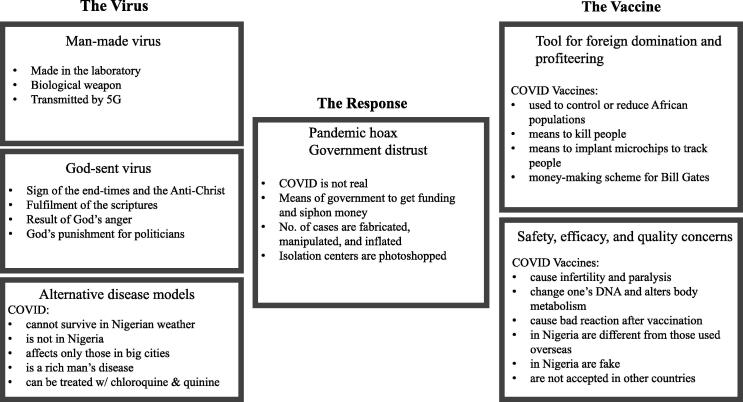
Conspiracy theories and misinformation about COVID-19 and the vaccines in Nigeria.

Rather than give participants a list of conspiracy theories or misinformation to choose from, we elicited their responses by asking what they had heard in their community or read about COVID-19 and the vaccines. We elicited a total of 33 different statements. It is noteworthy that each respondent had heard at least one conspiracy theory or piece of misinformation about the subject. Analysis of the statements they volunteered revealed three broad themes of the misinformation and conspiracy theories– statements about the nature of the virus, the motivations and validity of the pandemic response, and the vaccine products (Fig. 1).

More than half of the participants had heard misinformation or conspiracy theories about the virus itself. Some reported hearing the virus was manufactured in laboratories, was a biological weapon or resulted from 5G technology.

One respondent heard, *“COVID is not a natural disease; it was technologically developed” –* State program officer (KII).

The statements claiming the virus was man-made did not indicate who made them. In sharp contrast, other circulating narratives associated the viral origin with God and the Anti-Christ. For example, some participants said they had heard that the COVID-19 pandemic is a sign of the end times, a fulfillment of the scriptures, a result of God’s anger, a punishment for politicians from God, a plan of the devil, and a plot of the Anti-Christ. These apocalyptic interpretations based on the Christian text were reported by seven participants, a Christian religious leader, two male adults, and four health workers.

*“Religiously, some will tell you it is the fulfilment of the scripture, where, in the book of Deuteronomy chapter 28 God says any person or any group of persons, I am paraphrasing anyway, who lives in disobedience, God will send down incurable diseases among them; so, those who believe in such warnings will say; ah! this is the fulfilment of the scripture.” –* Health worker (KII)

Another subset of statements about the virus neither referred to nefarious networks creating the virus nor to God sending it but were alternative disease models of the COVID-19 experience in Nigeria. Nearly half of the participants heard statements that COVID-19 cannot survive the hot Nigerian weather, there was no COVID-19 in the country (though it exists elsewhere), or that COVID-19 was a disease of city dwellers or rich people. Some heard COVID-19 is just another form of malaria and that chloroquine was the treatment for it.

A second narrative thread revolved around the pandemic response. These included statements that the pandemic response is a fraud, that the government exaggerates the COVID-19 situation in the country, and that politicians are using COVID-19 as an excuse to siphon money from the Nigerian government. Related to this concept of a fraudulent response effort was the most frequently reported statement in all categories- that COVID-19 is not real, with beliefs that there were ulterior motives behind case reports.

*“It [COVID] is not in Nigeria; the government is just using it to swindle public funds. It is just malaria.”* –Male adult (KII)

Regarding the third theme of COVID-19 vaccines, a subset of such statements claimed the vaccine was a tool for foreign dominance and profiteering. Many participants heard that the COVID-19 vaccines would be used to control or reduce the African population and would be a means to implant microchips in people to track them. There were also statements about the vaccines serving as a mode of introducing the 666 mark of the Anti-Christ.

*“I am a Christian pastor, I am a Christian, and I also have followers under me…some of us thinkthat this COVID 19 vaccine is a way of introducing 666 controls on the world”*- Christian religious leader (KII)

The other subset of statements on the vaccines characterized them as lacking safety, efficacy, and quality, particularly those deployed in Nigeria. Participants reported hearing that the vaccines change people’s DNA, alter the body’s metabolism, and cause harmful reactions. They also heard that the vaccines used in Nigeria are different from those used in wealthier countries.

### 3.3 Sources of information about COVID-19 and the vaccines

In addition to asking participants about what they had heard, we also solicited information about the sources they rely on to get information about the virus and vaccines. Across all regions, radio, television, and social media were the most frequently reported sources of information (or misinformation) about COVID-19 and COVID-19 vaccines. Box 1 highlights other sources of information mentioned by participants and ranked by most cited. Most program managers and HCW perceived information from WHO, Nigeria Centre for Disease Control (NCDC), and National Primary Health Care Development Agency (NPHCDA) as reliable because it is evidence-based, and these agencies were responsible for handling the crisis. Most community members regarded information received from the radio and television as trustworthy, while a few thought the same about information from the WHO and NCDC. Most participants believed social media information is unreliable because it is subjective and open for anybody to publish.

“*We know that we are in the days of social media and fake news everywhere. Some information cannot be faulted, like some from international bodies like the World Health Organization. This information is research information and is from the horses’ mouth*”. –Religious leader (FGD)

Box 1 **Sources of information about COVID-19 and the vaccines in Nigeria.**

**Box 1.** Sources of information about COVID-19 and the vaccines in Nigeria.

Radio (25); Television (24); Social media (15); NCDC (9); Media (6); Text messages (5); Church (5); Whatsapp (4); Seminars (4); Mosque (4); NPHCDA Trainings (4); Posters (3); Newspaper (3); Journals (2); Government Trainings (2); Facebook (2); WHO (2); NCDC Text messages (2); WHO website (2); Workshops (2); Health workers (2); Phone calls (2); Community sensitization (1); Friends (1); Sensitization (1); Internet sources (1); Traditional media (1); Newspapers (1); News (1); Non-governmental organisation workshop (1); Fliers (1); Non-governmental organisation (1); Town crier (1); NPHCDA (1); Unicef (1); Youtube (1); Instagram (1); Centre for Disease Control Workshop (1); Family (1); Electronic media (1); Situation reports (1); Partners trainings (1); State Primary Health Care Development Agency trainings (1); Peers (1); Government (1); Phone (1); Town gatherings (1); Hospital (1); Trainings (1); Mass media (1); Community health center (1); Print media (1); WHO handbills (1); Public awareness (1); Word of mouth (1); Colleagues (1); News outlet (1); Research (1); Presidential Task Force (PTF) (1); Online sources (1); and Pamphlets (1).

### 3.4 Belief in conspiracy theories

We explored why people might believe in these theories and found that reasons vary by the type of misinformation or conspiracy theory people subscribe to. For example, participants reported that some people did not believe in the existence of COVID-19 because they had not had a personal experience with a COVID-19 case or death.

“*They don’t believe because, in a community, no one has contracted it*.” – Religious leaders (FGD)

“*Because they have not seen any impact of the COVID. We are in this [community]…. we are more than one thousand people, and one day, they have never seen even one person affected by COVID”.* –Male Adult (KII)

Furthermore, those considering Nigeria’s tropical climate too hot for the virus to survive tended to believe there was no COVID-19 in Nigeria. The belief that the pandemic response was a fraud, and a means for official graft, was rooted in the concurrent denial of the existence of the virus and distrust of the government’s integrity and intentions.

*“Actually, the society at large, the people, do not trust the government. The system is surely corrupt. Though I believe it [COVID] exists, I have not seen anybody that has died because of it*… *Abroad, there is transparency, but here, they [government] just do it to get financial assistance from monetary bodies. At the same time, if you look at the society, I have not heard a single person having the COVID”.* – Religious leader (FGD)

In contrast, we found that those who reject the notion that COVID-19 is not in Nigeria or accept that COVID-19 is real have had personal experience with COVID-19 or seen or heard about someone affected or died due to the disease. When asked about their perception of COVID-19, most participants said they believe COVID-19 is real, and few said they do not.

*“I do [believe COVID is real]. Because we have lost so many people due to COVID, many people have been isolated and become unhealthy; that’s why I believe in it”.* –Pregnant woman (KII)

Furthermore, participants’ belief in the reality of COVID-19 was linked to perceived susceptibility to contracting COVID-19, the global impact of the virus, and the scale of the response, including national lockdowns.

“*Initially, people were trying to convince us that this thing is not real, that the government is just using it to siphon money. At a time, I started to believe, but I discovered that the whole world was still. I believe because if it is not real, the whole world will not stand still*”. –Male Adult (FGD)

Participants offered various characteristics when asked about the typical believers and supporters of conspiracy theories and the misinformation about COVID-19. These included: being uneducated or religious, being young with access to information, being elderly with no access to traditional and social media, and having not known or seen someone affected or killed by COVID-19. Some cited those in the Southeast who distrust the present political leadership and those in the North who reject Western interventions.

*“The belief of Igbo is different. The Igbo people do not believe whatever [the President] says”. -* Religious leader (FGD)

*“Basically, those that do not trust anything Western are the hardcore Muslims, not the regular Muslims but hardcore Muslims. Then those that do not believe the government tends to be the Igbo speaking part of our country”. –* Program manager (KII)

We inquired into how belief or sympathy for conspiracy theories may affect the intention to accept a COVID-19 vaccine. While some said it would not affect their intent to take the vaccine, others believed it would, given the circulating misinformation.

*“…I have a lot of things I am considering. I still have conflicting information about COVID-19 vaccines so, I am still watching to see what will happen. I believe in vaccines, but there is more information that we stumble on these days, and they make you go thinking”*. –Religious leader (FGD)

*“This is where the issue is. With the current spread of the news, or let me say rumor, it has made me believe that the vaccine is harmful, and they want to use it to reduce the population of Africa. Presently, I am privileged to be part of some Christian gathering that think there is a plan, and the vaccine they are planning to bring down here is not the same as that of the one over there. That is why they want the leaders to take it first and observe if anything does not happen; we will now know what to do”*. –Male adult (KII)

### 3.5 Perceptions about COVID-19 vaccines versus other vaccines (and immunization more broadly)

To examine the extent to which hesitancy around COVID-19 vaccines was distinct from or part of broader vaccine hesitance, we looked comparatively at statements about other vaccines and vaccination programs. The overwhelming majority of participants (98%) viewed maternal and childhood vaccines in the immunization schedule favorably due to the positive track record of eradicating diseases and preventing morbidity and deaths, especially among children.

“*Our children suffer much before because there is no vaccination*… *we now find it easier to take care of our children due to vaccination*”. – Pregnant women (FGD)

In contrast, participants’ opinions about the COVID-19 vaccines varied. Nearly half of the participants had concerns about the COVID-19 vaccines. For example, community members were concerned about vaccine efficacy and safety due to reported side effects and unease about the short period of vaccine development. Other concerns raised were that the vaccines are not needed in Nigeria because COVID-19 is not real, and they had not seen an infected person. It was also perceived that the African continent has a low death rate from COVID-19.

*“The rumor is why are they centered on bringing the vaccine here when there is a low death rate in Africa compared to other Western countries like America, where their death rate is very high. Let them use it to cure themselves because we have a low death rate here. Also, the rate at which they produced the vaccine within a short period of time when they said it should take at least three years to produce a vaccine. Is the vaccine to cure, prevent or even kill, we don’t know?”.* –Male adult (FGD)

Likewise, the lack of information on the duration of the vaccine’s protection, perception that the government is not transparent about the vaccines, and poor communications about the threat of COVID-19 to the public were mentioned as concerns. Furthermore, participants also felt that not knowing the timing of the vaccine availability and where to access the vaccine was problematic.

Some health workers and program managers also shared the same view, including concerns about vaccine storage capacity due to insufficient power.

*“Well, we are still having a little doubt because they are saying it is 50–50, that is 50% effective and 50% not effective”*.

*“Yes, I have an opinion because it is like they need to enlighten us because so many theories are coming out concerning the vaccine, of which now if they bring the vaccine, I doubt if I can take it. Because honestly, I need to know the effectiveness of the vaccine, how safe it is; you know there are so many stories concerning it”*. - Health workers (FGD)

Nevertheless, about half of the participants had favorable feelings towards the COVID-19 vaccines. Many of them were program managers and health workers who perceived the vaccines as safe and effective in protecting against COVID-19 and trusting the government to introduce safe and effective vaccines.

## 4 Discussion

Our study aims to collate and categorize the circulating conspiracy theories and misinformation around COVID-19 and COVID-19 vaccines in Nigeria to inform evidence-based communication messaging and intervention strategies. To our knowledge, this is the first thematic analysis of circulating conspiracy theories and misinformation about COVID-19 elicited through a qualitative inquiry from HCWs and adults in Nigeria. We found that exposure to unsubstantiated claims about COVID-19 was universal among our participants, confirming the ubiquity of these false narratives. The range of statements reported in our study is similar to a list of globally circulating misinformation and conspiracy theories about COVID-19 compiled by Nyika et al. [20]. The similarity indicates the borderless and far-reaching spread of these false claims that penetrate online and offline communities and can undermine people’s confidence in the COVID-19 response and vaccination.

In our study, the leading conspiracy theory was that SARS CoV-2 does not exist; therefore, the pandemic response is a means for the Nigerian government officials and politicians to obtain pecuniary gains from misusing the response funds. This view confirms similar skepticism reported in other studies and countries about the virus being perceived as a hoax and the COVID-19 prevention efforts as an avenue for politicians to embezzle public funds [21], [22], [23], [24], [25], [26]. Hoax-related conspiracy theories have been shown to predict refusal to engage in preventive behaviors, including vaccination [25]. A striking finding from this study is that the Igbos from Southeast Nigeria were perceived to have the highest level of skepticism about COVID-19. This is because of a distrust of the current government. The recent separatist movement in the Southeast has put the zone in the federal government’s crosshairs, creating a trust deficit and geopolitical tension that may now be spilling over into public health. National surveys have reported that the lowest rates of intention to receive the COVID-19 vaccine were among Southeast participants compared to those from other zones in the country [27], [28]. These low rates raise fundamental concerns that high COVID-19 skepticism may translate into vaccine hesitancy among Southeasterners who have historically had one of the highest rates of childhood vaccine uptake, who also show strong faith in routine childhood vaccines in our study. Nevertheless, we posit that as more cases and deaths become evident, the claim of COVID-19 as a hoax may have less salience to those who need the evidence to convince them. However, programs must also brace for those with lingering doubts, who will continue to perceive that the pandemic is exaggerated because SARS-CoV-2 has affected Africa much less than the United States, Europe, or India.

The second striking finding is the difference in the religious interpretation of the pandemic. While our study did not report any statement linking the pandemic with Islamic text or teaching, we found that a widespread understanding of the pandemic among Christians relates it to the end-times and the Antichrist predicted in Christian scriptures. These statements have been associated with prominent Nigerian pastors with strong followership [29] and with Christian evangelicals globally [30]. Interestingly, in previous experience of polio vaccine conspiracy and hesitancy in Nigeria, misinformation about vaccines and fertility was prominent among Muslim rather than Christian groups. This underscores the importance of engaging broadly with different population subgroups, not just those with historic hesitancy, to assess concerns and craft messaging accordingly.

In countries like Nigeria, where most people have a religious identity, religious leaders can be a powerful source of misinformation. Statements linked to religious teachings are difficult to refute or debunk by public health experts because these statements are based on beliefs rooted in faith commitments that cannot be directly addressed by scientific evidence. Therefore, religious leaders are vital to addressing religious-themed claims that undermine vaccine acceptance. Studies have shown that sensitizing and engaging religious leaders increased the acceptance and uptake of novel health interventions in Nigeria [31], helped to stop the Ebola epidemic in West Africa [32], and addressed misconceptions about polio vaccines that increased vaccine coverage in India and Pakistan [33]. Religious leaders must be engaged in the fight against COVID-19.

Statements asserting COVID-19 vaccines are tools of Western actors to control or reduce the African population, track people, and alter their DNA were other commonly reported conspiracy theories in our study, as well as in other studies [34], [35], [36]. These unsubstantiated claims may hinge on long-held concerns about a structural imbalance between Africa and the Western world, as well as the colonial legacies the continent continues to bear.

Public trust in the vaccine and its foreign producers, the government, and the health system delivering the services is critical for vaccination acceptance[2], [37]. Political leaders’ disparaging statements about the vaccines, such as Tanzania’s late President John Magufuli, which referred to the vaccine as “dangerous for our health” [36], are highly damaging and confusing for a skeptical public. Although some conspiracy theory beliefs are rooted in people’s distrust of the government and its institutions, these same actors have a vital role in engaging the public, building their confidence in the vaccine programs, and communicating the right messages [38], [39]. It is noteworthy that information from public health agencies and experts such as WHO, NCDC, and NPHCDA were seen by our participants as trusted. In other studies, trusted information from public health agencies has been associated with a lower tendency to believe in conspiracy theories[22], [38], [40], [41], [42].

The medium of communicating these messages is important. We found a high level of trust in information from radio and television among community members. This finding is similar to other studies that reported that obtaining COVID-19-related information through traditional media sources is associated with lower beliefs in conspiracy theories and misinformation narratives [22], [41], [43]. Conversely, exposure to digital media was associated with greater belief in conspiracy theories and misinformation [41], [44], [45]. A global survey reported that 88% of Nigerians cited social media as a source of their distrust. The survey also indicated that only 41% had seen fake news while using traditional media sources, and 47% sometimes believed the fake news they saw [46]. So, despite the allure and ease of using social media for information dissemination, traditional media such as radio and television must continue to play a central role in communicating credible information about COVID-19.

Our study shows that vaccine safety and efficacy concerns were a major issue and a frequent topic of misinformation. Communication messages that highlight the low risk of side effects, and high efficacy of the vaccines, will therefore address these concerns and promote vaccine acceptance. Recent evidence from ten LMICs, including Nigeria, support this assertion [47]. The study shows that respondents who were asked to consider a hypothetical vaccine with a 5% probability of side effects and 75% efficacy reported an acceptance rate of 67% or higher [47].

Finally, the current communication plans such as zonal town hall meetings, press briefings, text messages, social media posts, short videos, and voice notes aim to educate the public and encourage COVID-19 vaccine uptake as vaccines are rolled out. But, the strategy has had only a modest effect on the desired behavioral change [48]. Adaptive strategies and messages that counter the prevailing false narratives and conspiracy theories with community involvement are needed to build confidence about the COVID-19 vaccine and encourage acceptance.

### 4.1 Limitations

Our study methodology relies on a small sample size to elicit COVID-19 vaccine misinformation and conspiracies theories, perceptions, and intentions about the COVID-19 vaccine. Thus, the findings are not generalizable; instead, they are unique to regions and populations with similar contexts as Nigeria. Despite this limitation, we present important results and messaging recommendations for different regions and sub-groups to improve COVID-19 vaccine acceptance in Nigeria and other comparable contexts.

## 5 Conclusion

Misinformation and conspiracy theories about COVID‑19 vaccination are evident globally. Distrust in the government regarding the pandemic response and suspicion of global actors’ motives regarding the vaccine, especially its effectiveness and safety, is strongly associated with hesitancy. Thus, adaptive strategies and messages are needed in the face of vaccine hesitancy. Targeted awareness campaigns and messages that deliver transparent information about COVID-19 vaccine safety and efficacy and the development process are also needed to encourage vaccine acceptance.

## Funding

This study work was supported by 10.13039/100010269Wellcome Trust [Grant Number: 21556/Z/20/Z] to the Johns Hopkins Berman Institute of Bioethics and implemented by Direct Consulting and Logistics. Wellcome Trust was not involved in the study design, data collection, analysis and interpretation, report writing, and the decision to submit the article for publication.

## Data statement

All datasets generated and analyzed during the study are available from the corresponding author upon request.

## Authors’ contributions

CW led the study development and design, supervised the project implementation, supported data analysis and interpretation, and produced the second and final draft of the manuscript. COJ supported the study design, supervised the project implementation and data collection, led the analysis and interpretation, and developed the first draft of the manuscript. RF, RK, and CK contributed to designing the study, helped with interpreting the analysis, and critically revised the manuscript for important intellectual content. FA, SCKU, TB conducted the data collection and supported the data analysis. RGA supported the data interpretation and critically revised the manuscript for important intellectual content. EFJ supported the study design and data interpretation. All authors approved the final version for submission. RF served as the Principal Investigator, with CW, RK, CK, and COJ as co-investigators.

## Declaration of Competing Interest

The authors declare that they have no known competing financial interests or personal relationships that could have appeared to influence the work reported in this paper.
