# Supplementary material for: Conspiracy theories and misinformation about COVID-19 in Nigeria: Implications for vaccine demand generation communications
Source: Vaccine. 2022 Mar 18;40(13):2114–21. doi: 10.1016/j.vaccine.2022.02.005 (PMC8830779; doi:10.1016/j.vaccine.2022.02.005)
Supplement: Supplementary data 1 [file mmc1.docx]

Supplementary Material 1: Summary of the selected states and the selection criteria

| **State** | **Zone** | **Number of COVID-19 cases^a^** | **Unusual picture^b^** | **Security compromised** |
| --- | --- | --- | --- | --- |
| Cross River | South South | 0 | Yes | No |
| Ebonyi | South East | 5 | No | No |
| FCT | North Central | 316 | No | No |
| Gombe | North East | 103 | No | No |
| Kano | North Weat | 427 | No | No |
| Lagos | South West | 1308 | No | No |

^a^ Number of COVID-19 cases as of May 6, 2020.

^b^ States where the pandemic’s potential was underrated, and tests were not conducted early on in Nigeria’s pandemic
