# Supplementary material for: Conspiracy theories and misinformation about COVID-19 in Nigeria: Implications for vaccine demand generation communications
Source: Vaccine. 2022 Mar 18;40(13):2114–21. doi: 10.1016/j.vaccine.2022.02.005 (PMC8830779; doi:10.1016/j.vaccine.2022.02.005)
Supplement: Supplementary data 2 [file mmc2.docx]

Supplementary Material 2: Description of the six study sites

Cross River, located in the south-south region of the country, presented a peculiar context during the first four months of its pandemic. It was the last of the states in Nigeria to declare COVID-19 cases due to controversies around testing in the state. The first case was confirmed on July 6, 2020; Cross River has since recorded 334 COVID-19 cases and 17 deaths. The state also adopted lockdown measures, limiting mass gatherings and school closures to contain the pandemic’s spread. Response efforts are being coordinated by the state COVID-19 task force headed by the state commissioner of health

Ebonyi is located in the southeastern geopolitical zone of the country. There have been 1928 COVID-19 cases and 31 deaths as of March 4, 2021. Lockdown of non-essential services, closure of schools, ban on interstate travel, and market operations changes are key containment measures implemented in the state to contain the virus.

FCT, Abuja, remains the state with the highest COVID-19 cases in the north-central zone after Lagos state. As of March 4, 2021, there had been 19292 cases and 149 deaths in the state. To mitigate the pandemic’s spread, measures such as lockdown, school closure, a limit on mass gathering, a ban on interstate travels and curfews were also put in place. The state COVID-19 task force is coordinating COVID-19 response efforts under the leadership of the FCT Minister.

Gombe, a northeastern state, has recorded 2009 COVID-19 cases and 43 deaths as of March 4, 2021. There were also the closure of schools, a limit on mass gatherings, changes in market operations, and a ban on interstate travel to contain the spread of COVID-19 cases in the state. A COVID-19 state task force was also inaugurated under the leadership of the State Commissioner of Health.

Kano, located in the country’s northwest region, saw a rapid spike of COVID-19 cases within the first hundred days after the first case was reported in Nigeria. By the end of May 2020, Kano was classified as one of the COVID-19 high burden states. As of March 4, 2021, Kano has recorded a total of 3790 cases and 104 deaths. Kano’s containment measures included an interstate travel ban, a lockdown of non-essential services, and school closure. The Deputy Governor of Kano is leading the state COVID-19 task force.

Lagos is located in the southwestern geopolitical zone of the country. As of March 4, 2021, Lagos has recorded a total of 56132 cases and 410 deaths. It is the pandemic’s epicenter, having registered the highest number of cases. Containment measures adopted to limit the disease’s spread in the state included lockdown measures, school closure, a limit on mass gathering, a ban on interstate travels, and curfews. The State COVID task force coordinates Response efforts under the state Governor’s leadership and the Commissioner of Health.
