# Supplementary material for: Conspiracy theories and misinformation about COVID-19 in Nigeria: Implications for vaccine demand generation communications
Source: Vaccine. 2022 Mar 18;40(13):2114–21. doi: 10.1016/j.vaccine.2022.02.005 (PMC8830779; doi:10.1016/j.vaccine.2022.02.005)
Supplement: Supplementary data 3 [file mmc3.docx]

Supplementary Material 3: Qualitative interview list of participants, interviews conducted, and the number of participants interviewed.

| **Participant type** | **No. and interview type** | **No. of respondents** |
| --- | --- | --- |
| Fathers of children under five years of age | 1 FGD | 7 |
| Mothers of children under five years of age | 1 FGD | 7 |
| Pregnant women | 2 FGDs | 14 |
| Male youths | 2 FGDs | 14 |
| Female youths | 2 FGDs | 14 |
| Older male adults | 2 FGDs | 14 |
| Older female adults | 2 FGDs | 14 |
| Male adults | 2 FGDs | 14 |
| Female adults | 2 FGDs | 14 |
| Healthcare workers at PHC (i.e., Health post, Dispensaries and PHC facilities) level | 1 FGD | 7 |
| Healthcare workers at secondary (i.e., General and specialist hospitals) or tertiary (i.e., University Teaching Hospitals or Federal Medical Centers) care level | 1 FGD | 7 |
| Christian religious leaders | 2 FGD | 14 |
| Muslim religious leaders | 2 FGD | 14 |
| LGA program manager | 6 KIIs | 6 |
| State program manager | 6 KIIs | 6 |
| Health workers at PHC levels | 6 KIIs | 6 |
| Health workers at secondary or tertiary levels | 6 KIIs | 6 |
| **SUB-TOTAL** | **22 FGDs**  **24 KIIs** | **154**  **24** |
| **GRAND TOTAL** | **46** | **178** |
